# Cortical signatures of auditory object binding in children with autism spectrum disorder are anomalous in concordance with behavior and diagnosis

**DOI:** 10.1371/journal.pbio.3001541

**Published:** 2022-02-15

**Authors:** Hari Bharadwaj, Fahimeh Mamashli, Sheraz Khan, Ravinderjit Singh, Robert M. Joseph, Ainsley Losh, Stephanie Pawlyszyn, Nicole M. McGuiggan, Steven Graham, Matti S. Hämäläinen, Tal Kenet

**Affiliations:** 1 Department of Speech, Language, & Hearing Sciences, Purdue University, West Lafayette, Indiana, United States of America; 2 Weldon School of Biomedical Engineering, Purdue University, West Lafayette, Indiana, United States of America; 3 Department of Neurology, Massachusetts General Hospital, Boston, Massachusetts, United States of America; 4 Department of Radiology, Massachusetts General Hospital, Boston, Massachusetts, United States of America; 5 Athinoula A. Martinos Center for Biomedical Imaging, Massachusetts General Hospital, Charlestown, Massachusetts, United States of America; 6 Boston University School of Medicine, Boston, Massachusetts, United States of America; Newcastle University Medical School, UNITED KINGDOM

## Abstract

Organizing sensory information into coherent perceptual objects is fundamental to everyday perception and communication. In the visual domain, indirect evidence from cortical responses suggests that children with autism spectrum disorder (ASD) have anomalous figure–ground segregation. While auditory processing abnormalities are common in ASD, especially in environments with multiple sound sources, to date, the question of scene segregation in ASD has not been directly investigated in audition. Using magnetoencephalography, we measured cortical responses to unattended (passively experienced) auditory stimuli while parametrically manipulating the degree of temporal coherence that facilitates auditory figure–ground segregation. Results from 21 children with ASD (aged 7–17 years) and 26 age- and IQ-matched typically developing children provide evidence that children with ASD show anomalous growth of cortical neural responses with increasing temporal coherence of the auditory figure. The documented neurophysiological abnormalities did not depend on age, and were reflected both in the response evoked by changes in temporal coherence of the auditory scene and in the associated induced gamma rhythms. Furthermore, the individual neural measures were predictive of diagnosis (83% accuracy) and also correlated with behavioral measures of ASD severity and auditory processing abnormalities. These findings offer new insight into the neural mechanisms underlying auditory perceptual deficits and sensory overload in ASD, and suggest that temporal-coherence-based auditory scene analysis and suprathreshold processing of coherent auditory objects may be atypical in ASD.

## Abstract 2

To navigate everyday environments, the auditory system must analyze the temporal coherence of sound elements scattered across different frequencies to organize them into discrete perceptual objects. This neuroimaging study reveals that this process is altered in autism, potentially explaining the often-experienced sensory overload.

## Introduction

Successful navigation of environments with multiple stimuli fundamentally relies on the brain’s ability to perceptually organize the barrage of sensory information into discrete coherently bound objects on which cognitive processes such as selective attention can act. Failure of this scene-segregation process, where one source stands out as the foreground “figure” and the remaining stimuli form the “background,” can result in an overwhelming sensory experience that makes it difficult to select a source of interest while suppressing the others [1,2]. For both visual and auditory processing, temporal coherence across assemblies of neurons that code for different stimulus features is thought to promote binding of those features into coherent perceptual objects [3,4]. A leading hypothesis about sensory processing abnormalities in autism spectrum disorder (ASD) is that this kind of temporal “synthesis” of sensory information is atypical [5–8]. This hypothesis stems from behavioral data indicating that individuals with ASD often show impaired processing of dynamic stimuli, such as the coherent motion of visual dots [9]. In the auditory domain, where stimuli are naturally dynamic, atypical sensory-stimulation-driven behaviors, particularly the experience of sensory overload and difficulty with being able to selectively listen to a foreground sound source of interest, are ubiquitous in ASD. These deficits are most acutely expressed in complex environments with multiple stimuli, where separation of foreground speech from background noise is essential [10–12]. Although many lines of behavioral evidence in ASD are consistent with the impaired temporal synthesis hypothesis, direct neural correlates of such deficits have not been identified. In the visual domain, atypical neural responses to illusory figures and contours have been interpreted as indirect evidence of impaired figure–ground segregation in ASD [13–15]. However, it is not known whether similar processes may also underlie the widespread auditory processing abnormalities in ASD [10–12,16–22], which in turn may contribute to the well-documented speech processing and language impairments associated with the disorder [21,23–31].

**Fig 1:**
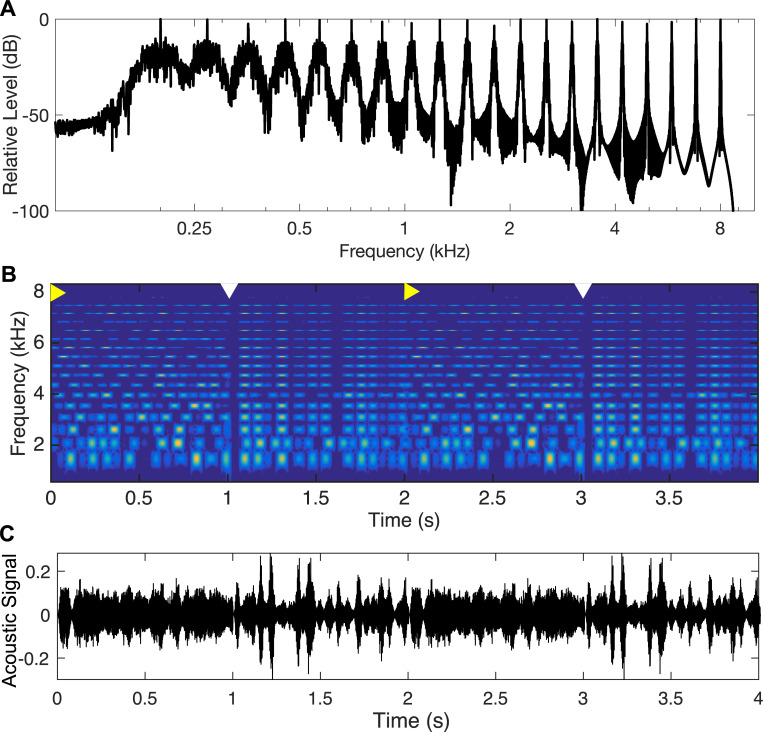
Stimulus properties and design. (A) Tone mixture used as carrier for the temporal coherence stimuli. (B) Depiction of one trial. Yellow right-pointing arrows on top mark onset of non-coherent modulation periods (1 s), and white down-pointing arrows on top mark onset of coherent modulations of *N* = 6, 12, or 18 out of 20 tones, randomized. There were two 1-s-long coherent periods per trial. (C) Sample acoustic waveform from one trial.

Here, we investigated whether auditory temporal coherence processing, and thus auditory figure–ground segregation and/or suprathreshold processing of coherent figures, is impaired in ASD, by employing a novel auditory paradigm. By manipulating temporal coherence in the scene with synthetic sounds, the paradigm was designed such that the acoustic features of the stimulus perceptually bind together into auditory objects with different levels of salience, with the salience of the foreground “figure” object increasing with increasing temporal coherence. More specifically, the stimulus consisted of a compendium of 20 different tones that were spaced in frequency such that they would excite equally spaced sections along the cochlea, following prevailing models of tonotopy in the human auditory system. The individual tones (Fig 1A) were amplitude modulated using a random envelope, with the envelope fluctuations being statistically independent, i.e., temporally incoherent, across the tones at the start of the stimulus. At set intervals through the stimulus time course, a subset of *N* tones out of the 20 (*N =* 6, 12, or 18) were switched from being modulated independently to being modulated with temporally coherent envelope fluctuations (Fig 1B). At this point, the stimulus percept would change from a large number of buzzing tones to a broadband irregular click-train-like sound popping out as a coherent auditory object from the background buzzing. A key feature of the stimulus design was that the frequency spacing between any 2 tones was 50% larger than the bandwidth of cochlear filters, so that the different tones would excite mostly distinct tonotopic sections of the ascending auditory pathway. Consequently, robust responses to increases in temporal coherence would need to arise primarily from downstream neural computations that combine information across neurons driven by different tonotopic channels. As the number of tones that switched from being incoherent to coherent increased, the perceptual pop-out would become more salient, and would be expected to elicit increasingly larger neural responses. Sample stimuli for all 3 coherence levels are available in S1–S3 Audios. Note that our stimulus paradigm deliberately maintains fixed modulation statistics within each tonotopic channel throughout the length of the stimulus, thus avoiding discontinuities within any given channel, and deviating from the more classic design pioneered by Teki et al. [32–34]. We chose to do this in spite of the fact that this meant coherent periods might exhibit an increase in overall amplitude, because cochlear tonotopic processing dictates that the central nervous system does not have access to the overall amplitude and instead is driven by individual tonotopic channels. Thus, any neural responses that reflect the overall amplitude would also have to arise from neural computations that use the temporal coherence to combine information across different tonotopic channels. A supplementary behavioral experiment was also conducted to validate this design.

To quantify the cortical processing of auditory temporal coherence, we recorded magnetoencephalography (MEG) signals from 26 typically developing (TD) and 21 ASD participants matched on age (7–17 years), gender, and IQ, using the above paradigm. The stimulus was presented passively while the children were watching a silenced, no subtitles, video of their choice. The participants were instructed to ignore the sounds they heard, so as to minimize potential differences due to attention across the 2 groups, and to focus on group differences in low-level auditory processing. We hypothesized that children with ASD would show reduced growth of the cortical response with increasing salience of the foreground object, and thus atypical neural signatures of temporal coherence processing.

## Results

### Source localization and evoked responses

**Fig 2:**
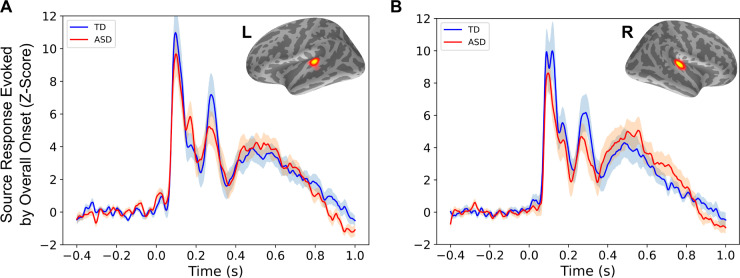
Responses evoked by overall stimulus onset. (A) Averaged left hemisphere evoked responses relative to stimulus onset, in source space, based on individually identified regions of interest (inset), for each group. (B) Same as (A), for the right hemisphere. Shaded areas show standard error per group. Underlying data can be found on Zenodo (doi: 10.5281/zenodo.5823656). ASD, autism spectrum disorder; L, left; R, right; TD, typically developing.

The MEG signals at the sensors were used to estimate neural source currents on the cortex using individual structural magnetic resonance imaging (MRI) data. As expected, in all participants, the averaged evoked responses relative to the overall onset of the stimulus (yellow rightward-pointing arrows in Fig 1B) were localized to the early auditory cortex in both hemispheres. Within these left and right regions of interest (ROIs), there were no significant group differences or tendencies towards group differences in either amplitude or latency of the evoked responses, in either hemisphere, across all identified peaks (Fig 2). All subsequent results are reported based on data from these individually derived ROIs. Because no other brain areas showed a consistent response across all participants, all of our analyses were limited to these ROIs.

The responses evoked by the changes in temporal coherence (white downward-pointing arrows in Fig 1B), rather than the responses evoked by the overall stimulus onsets, are of primary interest to the question of figure–ground segregation. There were no significant differences in the responses evoked by these coherence changes between the right and left hemispheres for either group (S1 Fig), and therefore results from both hemispheres were combined for all subsequent analyses.

### Auditory cortex responses evoked by onset of stimulus coherence

**Fig 3:**
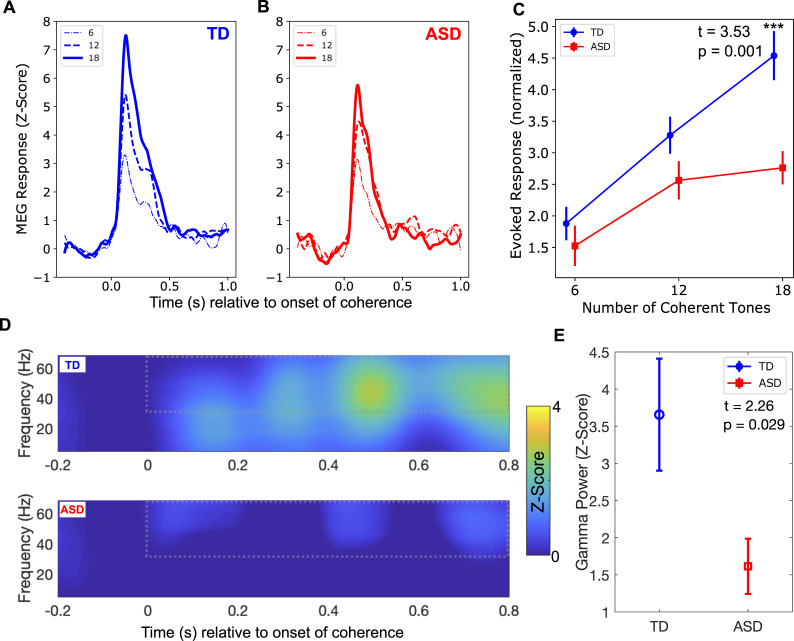
Evoked and induced responses following changes in temporal coherence. (A) Evoked responses in the typically developing (TD) group, where time = 0 represents the onset of a 1-s coherently modulated period within the trial (white arrows in Fig 1B), not the onset of the trial. The responses are plotted for each of the 3 coherent-modulation conditions (*N* = 6, 12, and 18), each averaged separately. (B) Same as (A), for the autism spectrum disorder (ASD) group. (C) Averaged amplitude of the responses in (A) and (B), over the 50-ms to 450-ms time window, for each condition and group. ***The group difference was significant at the *N* = 18 condition. (D) Time-frequency plot for the TD group (top) and ASD group (bottom) in response to the *N* = 18 condition, where time = 0 represents the onset of the 1-s coherently modulated period within the trial (white arrows in Fig 1B), not the onset of the trial. Dotted line outlines the time-frequency window used to compare the two groups. (E) Group difference within the time-frequency window outlined in (D). For both the evoked response and the induced response analysis, the magnetoencephalography (MEG) data during the incoherent stimulus portion that preceded the onset of the coherent figure was used as the baseline. Underlying data can be found on Zenodo (doi: 10.5281/zenodo.5823656).

At the onset of the coherent portion of the stimulus (white downward-pointing arrows in Fig 1B), a robust auditory evoked response was observed for all 3 levels of stimulus coherence, in both the TD (Fig 3A) and ASD (Fig 3B) groups, with both groups showing an increase in the magnitude of the response as the percentage of coherent tones increased (6, 12, and 18 out of 20), in line with the increasing perceptual salience of the foreground figure. Note that the baseline relative to which these coherence-related responses were quantified was the incoherent portion of the stimulus (just before the downward-pointing white arrows in Fig 1B) and not the pre-stimulus silent periods. In the TD group, both the M100 (M1, 50–150 ms) and the M200 (M2, 250–450 ms) components of the response were reliably identifiable for most participants, while in the ASD group this was true only for the M1 component. Because the M2 peak was not discernable for most ASD participants (S2B Fig), for the remaining calculations we focused on the response in the combined M1 + M2 time window (50 ms to 450 ms in Fig 3A and 3B). Quantifying the magnitude of the response within this time window showed that, overall, the growth in response magnitude with increasing coherence was significantly more sluggish in the ASD group than in the TD group (Fig 3C). The main effect of group (*F*[1,45] = 8.55, *p* = 0.005) and the group × condition interaction (*F*[2,90] = 3.61, *p* = 0.03) were both significant. The groups differed most in the *N =* 18/20 tones condition (*t*[45] = 3.53, *p* = 0.001).

Another component of the cortical response to temporal coherence is the induced gamma band (>30 Hz) activity, which has been associated with perceptual figure–ground segregation in vision [35]; with processing of complex sounds such as speech in audition [36,37], particularly with active listening [38]; and with temporal synthesis of information via predictive coding [39]. Gamma band activity is also thought to be mediated by inhibitory (GABAergic) mechanisms [40], which have been documented to be abnormal in ASD [41–50]. In the *N =* 18/20 coherent tones condition, the TD participants had increased gamma band power (30–70 Hz), especially during the later period (400 ms onwards) of the response (Fig 3D, top), while no such increase was observed in the ASD group (Fig 3D, bottom). Indeed, the gamma band power was significantly lower in the ASD group (Fig 3E). Thus, not only was the response evoked by the object formation event reduced in ASD, but neural oscillations induced by the salient auditory figure were also reduced.

### Classification by diagnosis and correlations with behavioral measures

**Fig 4:**
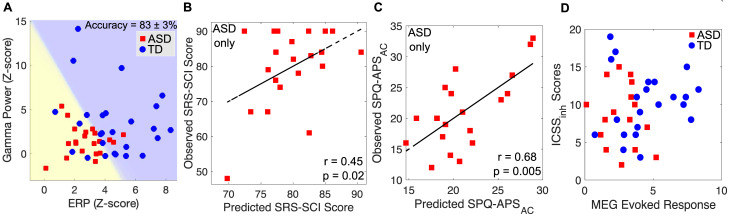
Behavioral correlations and group classification. (A) Group classification, using the individual evoked responses and induced gamma band activity as the inputs to the model. Yellow background depicts predicted ASD diagnosis. (B) The behaviorally assessed Social Responsiveness Scale–Social Communication and Interaction (SRS-SCI) scores, plotted against the same scores predicted by the model constructed using individual evoked responses and induced gamma band activity. (C) Same as (B), for the age-corrected Sensory Profile Questionnaire–Auditory Profile Subscale (SPQ-APS_AC_) scores. (D) The averaged evoked responses for the *N* = 18 tones condition within the trial, plotted relative to the NEPSY-II–Inhibition Inhibition Contrast Scaled Score–Inhibition (ICSS_inh_) behavioral measure, for each group. No correlation was observed between these measures. Underlying data can be found on Zenodo (doi: 10.5281/zenodo.5823656). ASD, autism spectrum disorder; MEG, magnetoencephalography; TD, typically developing.

To assess the relevance of the evoked responses and induced gamma band power to the ASD diagnosis, we used a blind linear support vector machine (SVM) classifier, with the individual *z-*scores for gamma band power and normalized evoked responses (both for the *N =* 18 coherent tones condition) as the predictive features. The classifier showed an accuracy of 83% ± 3% for group classification (Fig 4A).

We then examined the correlation between the cortical response and ASD symptom severity, using 2 behavioral measures: The Social Responsiveness Scale–Social Communication and Interaction (SRS-SCI) subscale, which assesses social-communicative symptoms, and the age-corrected scores (SPQ-APS_AC_) from the Sensory Profile Questionnaire–Auditory Profile Subscale (SPQ-APS), which assesses auditory processing abnormalities. To that end, we constructed linear models to predict the SRS-SCI and SPQ-APS_AC_ from the individual *z-*scores for the evoked responses and the gamma band power, and compared the predicted scores to the behaviorally measured individual scores. We found that the predicted scores for both the SRS-SCI (Fig 4B) and the SPQ-APS_AC_ (Fig 4C) were significantly correlated with the behavioral scores. Note that even without a correction for age, the predicted SPQ-APS still correlated with the observed SPQ-APS, just not as strongly (S3 Fig).

To quantify whether the observed effects might be attributable to gross variations in attentional control, we used data from the participants for whom the NEPSY-II–Inhibition Inhibition Contrast Scaled Score–Inhibition (ICSS-I) was collected. During the MEG session, participants were asked to attend to the movie (silenced, no subtitles) they were watching, and ignore the stimulus. Despite the passive nature of the measurements, the coherent auditory figure can elicit a pop-out effect, where attention is drawn in a bottom-up manner. It is possible that those participants less able to ignore the stimulus would also have stronger responses. The ICSS-I measures precisely this—the ability to inhibit attention. While there was no detectable group difference in ICSS-I between the groups, it is nonetheless possible that the ICSS-I would correlate with the magnitude of response (irrespective of diagnosis), should the response be driven in part by attention being drawn to the stimulus. We found no correlation between the ICSS-I and the magnitude of the MEG evoked responses in either group (Fig 4D), indicating that the group differences are likely due to lower-level auditory processes, as hypothesized, rather than to attention-driven processes. There was also no correlation between the ICSS-I scores and the MEG gamma band activity (S5 Fig).

Lastly, to test whether increases in overall amplitude during the coherent portions of the stimulus—rather than across-channel coherence relationships—could account for the observed group differences, we conducted a small supplementary behavioral experiment in a different group of adult participants (6 participants). In this experiment, the across-channel relationships were manipulated by changing the spectral separation between the coherent subset of tones for 2 coherence levels near threshold (either 4 coherent tones or 6 coherent tones). Crucially, these manipulations don’t change the overall amplitude. Yet, these manipulations had a large effect on figure detection, showing that across-channel relationships, and not overall amplitude changes, were the main factor driving figure–ground segregation (S4 Fig). These results are in line with the constraints imposed by cochlear tonotopic processing, confirming the validity of our stimuli for probing temporal-coherence processing that combines information across neurons responding to different tonotopic channels.

## Discussion

This study aimed to test whether the cortical correlates of auditory figure–ground segregation based on temporal coherence may be abnormal in ASD. As hypothesized, we found that children with ASD had significantly attenuated evoked responses to the pop-out of the foreground figure, alongside a lower magnitude of induced gamma band activity. Importantly, the cortical measures were not correlated with the behaviorally assessed ability to suppress attention, suggesting that lower-level auditory processes contribute to the observed abnormalities, rather than overall differences in attention to the pop-out of the auditory figure while being distracted by the movie. The cortical measures did, instead, correlate with both ASD severity, measured behaviorally, and abnormality of auditory sensory processing, also measured behaviorally.

These results suggest that neural processing of the temporal coherence of the acoustic constituents of an auditory stimulus is atypical in ASD. More specifically, the growth of the cortical neural response with increasing levels of temporal coherence was more sluggish in ASD. This is consistent with a scenario where the neurophysiological substrates that support the perception of highly temporally coherent complex sounds as salient foreground objects are impaired in ASD. Given the importance of temporal coherence as a binding and scene segregation cue in natural sounds, the atypical processing of temporal coherence in ASD could contribute to poorer object binding, as has indeed been suggested [51], and demonstrated indirectly in the visual domain in ASD [13–15]. In scenes with multiple sound sources, the reduced growth of the response with temporal coherence could lead to foreground sounds “standing out” less saliently, contributing to impaired auditory selective attention, i.e., “filtering,” and the experience of sensory overload [2]. Such abnormalities would inevitably also impact speech and language processing, which are highly temporally sensitive [36,52], especially in environments with multiple sound sources. Indeed, speech impairments in noisy environments in particular have been documented in ASD [11,53,54].

The pattern of evoked-response differences seen between ASD and TD (i.e., largest differences in the high-coherence condition) raises the possibility that suprathreshold salience of temporally coherent auditory figures, rather than threshold sensitivity to temporal coherence, may be impaired in ASD. In the visual domain, individuals with ASD show elevated thresholds for detecting (i.e., reduced sensitivity) coherently moving dots [55] and global patterns of movement of simple forms [9]. In the auditory domain, individuals with ASD show reduced suprathreshold ability to take advantage of coherent temporal fluctuations to derive masking release [12]. However, to the best of our knowledge, threshold sensitivity in ASD to temporal-coherence-based auditory pop-out has not been directly assessed. Our results showed that a combined M1+M2 MEG evoked response was measurable for all three coherence levels for both groups, suggesting suprathreshold rather than sensitivity deficits in ASD. However, the M2 peak *per se* was not discernable for a majority of ASD subjects at any of the three coherence levels. It is presently unknown how the M1 and M2 responses each relate to behavioral sensitivity to temporal coherence. Future behavioral studies would be needed to explore whether sensitivity or suprathreshold auditory coherence processing is anomalous in ASD.

While it is not possible to rule out the role of attention-based differences in contributing to the reduced growth of evoked responses in ASD, it is unlikely that attentional abnormalities are the primary driver of these differences. Tone-cloud stimuli similar to the sounds employed in this study have been used with functional MRI, MEG, and electroencephalography to probe auditory figure–ground segregation in typical adults. These studies showed that although evidence of temporal coherence processing is measurable in the auditory cortex, attention can significantly enhance the neural representation of the foreground figure, especially in later parietal regions [32,34,56,57]. Indeed, animal models show that temporal coherence sensitivity and neural computations that support auditory scene analysis begin subcortically, as early as the cochlear nucleus [58]. There is also evidence of reduced, rather than increased, attention in ASD to non-speech auditory stimuli, even when perception of these stimuli is identical between the groups [59]. By virtue of using a passive design and focusing the analysis on the auditory cortex, the results of the present study show atypical processing in ASD of even early aspects of temporal-coherence-mediated computations. Of course, this does not preclude the possibility that in an active auditory scene analysis task, impairments in later attentional processing in ASD would further exacerbate the perceptual deficits. Attention networks are also known to be anomalous in ASD [60]. Another factor that makes it less likely that deficits in attentional processing can fully account for the differences reported in this study is that we observed no gross differences in the evoked responses relative to the overall onset of the auditory stimulus; it is well known that attention modulates auditory evoked responses [61,62], and thus the absence of differences in this overall response is consistent with an absence of difference in attentional load for this particular paradigm. Furthermore, across participants in both groups, there was no correlation between ICSS-I score, which measures overall ability to inhibit attention, and MEG evoked responses. Thus, the results of our study suggest that pre-attentive aspects of auditory figure–ground segregation are already impaired in ASD.

In addition to sluggish growth of evoked responses in ASD, we also found that the induced gamma band activity that was observed in the TD group (in the high-coherence condition) was reduced in the ASD group. There are many possible interpretations for the observed gamma band differences. Computational models of gamma rhythms predict that these rhythms may be involved in coherence-dependent selective routing of sensory information to downstream regions [63]. Earlier in vitro studies using voltage-sensitive dyes, and more recent in vivo optogenetic studies, provide evidence that this coherence-dependent gating is mediated by GABAergic processing [40,64], which is known to be atypical in ASD [42–50]. However, unlike the evoked-response differences, these gamma band differences were observed later in time (400 ms and after) relative to the onset of coherent modulations. Thus, although the evoked responses likely reflect pre-attentive processing at least in part, the increase in induced gamma band activity in the TD participants may indicate attention capture. The lack of gamma band activity in ASD may thus indicate failure of attention capture, either as a consequence of the impaired coherence processing and reduced salience with which the foreground figure pops out, or because of differences in attentional processing in general in ASD [65]. Another possibility is that the gamma band activity may encode prediction errors associated with the temporal synthesis of information during predictive coding [39], which also may be impaired in ASD [66]. These results are consistent with our prior studies of ASD that also found evidence of abnormal local functional connectivity, as well as increased feedforward connectivity alongside reduced top-down gain control [67–69]. More targeted future work is needed to delineate the precise mechanisms that contribute to the gamma band differences observed in the present study.

Finally, our stimulus design deviated from the established stochastic figure–ground stimuli used by Teki et al. [32], and as a result, the overall amplitude of the stimulus was allowed to change during coherent periods. As discussed in the Results section, because of the cochlear constraints, it is unlikely that these overall amplitude changes themselves would drive differences in evoked responses. Furthermore, because many children with ASD exhibit hypersensitivity to more intense sounds, it has been hypothesized by many research groups that neural responses in ASD would be larger, not smaller, as stimulus amplitude is increased. Despite great interest in this question, surprisingly, there are no reports to our knowledge describing atypical auditory cortical responses as a function of stimulus intensity in children with ASD and average or above average IQ. Our own unpublished MEG data examined exactly this question, in a group of 11 TD children and 15 ASD children (average age 10 years, all with IQ > 80); to our own surprise, we found no indication of group differences in the cortical evoked-response amplitudes as a function of stimulus intensity (55 dB to 85 dB in 10-dB steps). The lack of published data on this question may indicate that other groups also did not see such differences, and therefore also left the data unpublished.

In summary, we have found that cortical evoked responses to increasingly coherent auditory stimuli show reduced growth of magnitude in children with ASD relative to TD children. The fact that the observed neurophysiological metrics correlated with behavioral measures that tap into individual ASD presentations, specifically the SRS-SCI and auditory processing scores, confirms the relevance of these neural measures to ASD. One key advantage of our novel stimuli and the passive design is that the paradigm is translatable to patient populations that are difficult to work with, where behavioral assessments cannot be performed. Along the same lines, the paradigm is also applicable to animal models of ASD and other neurodevelopmental disorders. In sum, our observations of reduced growth of neural responses with increasing temporal coherence of auditory stimuli in ASD provide novel insight into the mechanisms that ultimately result in the abnormal sensory and social-communicative processing deficits characteristic of ASD.

## Materials and methods

### Participants

Twenty-six TD school-aged children and 21 children with ASD participated in the study. The study was conducted in accordance with the principles expressed in the Declaration of Helsinki. Parents provided written informed consent according to protocols approved by the Massachusetts General Hospital Institutional Review Board (IRB protocol #2005P001768). Participants provided assent in addition to parent consent. Phenotypic data collected from the participants are summarized in Table 1. The age range in both groups was 7–17 years, with the mean age being 13.8 years and the median age being 14 years. All participants were right-handed, with handedness information collected using the Dean Questionnaire [70]. Hearing was assessed by testing cochlear sensitivity to sounds, and the groups were indistinguishable based on distortion-product otoacoustic emissions (see “Otoacoustic emissions” below). Participants with ASD had a prior clinical diagnosis of ASD and met ASD criteria on the Autism Diagnostic Observation Schedule, Second Edition (ADOS-2) [71], administered by trained research personnel who had established inter-rater reliability (with co-author RMJ). The Social Communication Questionnaire, Lifetime Version, was administered to rule out ASD in TD participants, and to further confirm ASD in ASD participants. ASD participants who did not meet a cutoff of ≥15 on the Social Communication Questionnaire, or had a borderline score on the ADOS-2, were then further evaluated by expert clinician co-author RMJ, to confirm the ASD diagnosis. Individuals with autism-related medical conditions (e.g., fragile X syndrome, tuberous sclerosis) and other known risk factors (e.g., gestation < 36 weeks) were excluded from the study. All TD participants were below the threshold on the Social Communication Questionnaire and were confirmed to be free of any neurological or psychiatric conditions, and free of substance use for the past 6 months, via parent reports and self reports.

**Table 1:** Characterization of the participants.

Characteristic	ASD (*N =* 21; 3 females)			TD (*N =* 26; 4 females)			*p*-Value
	*N*	Mean (SE)	Range	*N*	Mean (SE)	Range	
Age	21	14.0 (0.6)	7–17	26	13.6 (0.6)	7–17	0.70
Nonverbal IQ	21	103 (3.4)	59–130	26	112.1 (2.8)	93–147	0.05
Verbal IQ	21	102.6 (4.7)	61–141	26	117.3 (2.5)	98–144	0.009
ADOS-2	21	11.2 (0.8)	7–18	—	—	—	—
SCQ_Lifetime_	19	18.8 (1.8)	8–31	24	3.5 (0.7)	0–12	1 × 10^−8^
SRS-SCI	21	74.6 (2.5)	48–90	25	45.2 (1.1)	36–60	1 × 10^−11^
SPQ-APS	20	15.35 (1.0)	7–23	26	22.1 (0.5)	17–25	1 × 10^−6^
ICSS-I	16	8.62 (1.03)	2–15	22	10.32 (0.9)	3–19	0.224

The *p*-values are from 2-sample *t* tests for a difference in means between the ASD and TD groups. Autism Diagnostic Observation Schedule, Second Edition (ADOS-2): total score. Social Communication Questionnaire, Lifetime Version (SCQ_Lifetime_): total score. Social Responsiveness Scale–Social Communication and Interaction (SRS-SCI): subscale T-score. Sensory Profile Questionnaire–Auditory Profile Subscale (SPQ-APS): score for questions 1–7. NEPSY-II–Inhibition Inhibition Contrast Scaled Score–Inhibition (ICSS-I) [72].

Verbal IQ (VIQ) and nonverbal IQ (NVIQ) were assessed using the Kaufman Brief Intelligence Test, Second Edition (KBIT-2) [73], for 18 ASD and 17 TD participants, and using the Differential Ability Scales–II (DAS-II) [74] for 3 ASD and 9 TD participants. The 2 groups differed on both NVIQ and VIQ. The group difference in NVIQ was driven by a single ASD participant with a NVIQ of 59. When this participant was removed from the sample, the group difference was no longer significant. We therefore checked all the correlation results excluding this participant, and all of the significant correlations and classification results remained significant to a similar extent with and without this participant. Given these results, we kept the participant for all of the analyses, and did not correct for NVIQ. The group difference in VIQ is in line with the ASD phenotype, and therefore we did not correct for this difference. For the correlations between MEG data and behavioral scores, we focused on 2 ASD-related behavioral assessments. The first was the Social Responsiveness Scale, Second Edition (SRS-2) parent report [75], which was designed as a quantitative measure of autism-related symptoms. The SRS-2 yields separate subscale scores for social communication and interaction (SRS-SCI) and for restricted interests and repetitive behaviors (SRS-RRB). Confirmatory factor analyses demonstrate a 2-factor structure that differentiates the 4 SRS-SCI subscales from the SRS-RRB subscale [76]. Here, we focused on the SRS-2 SRS-SCI composite as a measure of ASD social symptom severity. The second ASD-related behavioral assessment was the Sensory Profile Questionnaire [10], and specifically the score from the auditory section of the sensory profile (SPQ-APS). Note that because we hypothesize that the processes probed here would correlate with sensory sensitivity or avoidance, rather than sensory seeking, we chose to exclude the response to question 8, which probes sensory seeking directly (“Enjoys strange noises/seeks to make noise for noise’s sake”), from the score. We also checked the results with question 8 included, and they remained significant, although the effect was slightly reduced.

### SPQ-APS age effects

Because of the wide age range, we checked whether any of the MEG responses or behavioral measures correlated with age. Of all of the considered MEG and behavioral measures, the only measure that was (weakly) correlated with age was SPQ-APS (S3A Fig; *p* < 0.04 within the ASD group). To account for this, we adjusted the SPQ-APS scores for age, and used the residuals to examine correlations with the MEG measures (Fig 3C). The age-corrected value is referred to as SPQ-APS_AC_ in the text. The correlations between the observed and predicted SPQ-APS values uncorrected for age are shown in S3B Fig.

### Otoacoustic emissions

To obtain an objective correlate of cochlear mechanical function, distortion-product otoacoustic emission (DPOAE) growth functions were measured as a function of the level of the f2 primary tone (f2 = 1, 2, 4, or 8 kHz) using an integrated probe with 2 independent sound sources and a low noise microphone (Etymotic ER-10C, Etymotic Research). The frequency and level of the f1 tone were varied according to the formula provided by [77] to maximize the level of the DPOAE for each level of the f2 tone. The DPOAE level was estimated at the distortion frequency of 2f1–f2. The number of trials was varied such that the noise floor (estimated by subtracting the mean of odd trials from even trials) was −15 dB SPL or better at 1 kHz and 2 kHz, and −25 dB SPL or better at 4 and 8 kHz. All participants had 2f1−f2 DPOAE amplitudes of at least 6 dB above the noise floor for L2 of 40 dB SPL, consistent with normal hearing. The TD and ASD groups were indistinguishable based on the DPOAE growth functions at each of the 4 tested frequencies.

### MEG data acquisition and preprocessing

MEG data were acquired inside a magnetically shielded room (IMEDCO) using a MEG 306-channel dc-SQUID Neuromag VectorView system (Elekta-Neuromag, Helsinki, Finland) with 204 planar gradiometers and 102 axial magnetometers. Two bipolar electro-oculogram (EOG) electrode pairs measured horizontal eye movements and blinks. A bipolar chest electrode pair was used to record electrocardiogram (ECG) data. All data acquisition was performed at a sampling rate of 3,000 Hz and subsequently downsampled to 1,000 Hz for analysis. After manual marking of bad channels during acquisition time, signal-space separation (SSS) was done to attenuate contributions from sources physically outside the radius of the helmet [78]. Four head position indicator coils were used to monitor head position. Samples containing artifacts associated with eye movements and blinks were extracted by detecting peaks from the vertical EOG channel; samples with cardiac artifacts were similarly identified from ECG data. These samples were used to define spatial filters to help suppress artifacts using the signal-space projection method: 2 filters for blink artifact removal and 2 for cardiac artifact removal. Data were then band-pass filtered to between 1 and 90 Hz. Data were then segmented into epochs time-locked to stimulus events. Epochs were rejected if the peak-to-peak range over the epoch exceeded either 1,000 fT in any magnetometer channel or 3,000 fT/cm in any planar gradiometer channel. Every stimulus condition included between 200 and 240 epochs for every participant included in the data analysis.

### Source localization

T1-weighted, high-resolution MPRAGE (magnetization-prepared rapid acquisition gradient-echo) structural images were acquired on a 3.0 T Siemens Trio whole-body MRI scanner (Siemens Medical Systems, Erlangen, Germany) using a 32-channel head coil. The in-plane resolution was 1 × 1 mm^2^, slice thickness was 1.3 mm with no gaps, and the repetition time/inversion time/echo time/flip angle values were 2,530 ms/1,100 ms/3.39 ms/7 degrees. The geometry of each participant’s cortical surface was reconstructed from the 3D structural MRI data using FreeSurfer software (http://surfer.nmr.mgh.harvard.edu). After correcting for topological defects, cortical surfaces were triangulated with dense meshes with about 130,000 vertices in each hemisphere. For visualization, the surfaces were inflated, thereby exposing the sulci [79]. The cortical surface was decimated to a grid of 10,242 dipoles per hemisphere, corresponding to a spacing of about 5 mm between adjacent source locations on the cortical surface. The watershed algorithm was used to generate the inner skull surface triangulations from the T1-weighted MR images of each participant. The MEG forward solution was computed using a single-compartment boundary element model (BEM) [80]. The head position information from the first run was used to estimate the sensor location relative to the source space. Sensor data from subsequent runs were transformed to correspond to the head position of the first run during the signal-space separation preprocessing step [78]. The cortical current distribution was estimated using minimum-norm estimate (MNE) software (http://www.martinos.org/mne); in this solution, we assumed that the orientation of the source was fixed and perpendicular to the cortical mesh. Measurement noise covariance was estimated from the pre-stimulus MEG data pooled across all conditions and runs. To reduce the bias of the MNEs toward superficial source distributions, we used a noise-normalization procedure to obtain dynamic statistical parametric maps (dSPMs) as *z-*scores [81].

We used the sensor-level evoked response combined across all conditions to localize the sources on the cortex; ROIs for analysis were defined based on individual-participant source localization results. No whole-brain group analysis was performed for this study. ROIs were defined based on the localization of the onset response evoked by the start of the auditory stimulus pooled across all conditions. The dSPM scores in the 90- to 140-ms time window following the onset of the auditory stimulus were averaged to yield a single *z-*score per pixel. This whole-brain *z-*score map was then thresholded such that 2 contiguous clusters remained, 1 per hemisphere. It was manually verified that these clusters overlapped with the anatomical FreeSurfer label containing the Heschl’s gyrus in each participant. Note that the point spread from MEG inverse imaging often yielded activations at adjacent sulci (as in Fig 1C). For each participant, the largest contiguous set of 32 vertices in the left and right hemisphere ROIs was then averaged to extract source time courses for analysis. Because each individual’s ROI-based time courses were self-normalized in the data analysis using *z-*scoring relative to the baseline fluctuations, a fixed ROI size was used rather than attempt to tailor the ROI size to each individual’s activation pattern. To more readily quantify the difference between the 2 groups, as well as to minimize the effects of noise, individual ROI time series were normalized based on the individual baseline fluctuations using *z-*scoring.

### Auditory stimulus

The primary goal of the stimulus design is that the temporal coherence between acoustic features should be amenable to parametric manipulation. Twenty tones were equally spaced from 200 Hz to 8 kHz on an “ERB scale” based on psychophysical estimates of cochlear tuning derived from a simultaneous masking paradigm [82] and were synthesized such that each tone had an intensity of 60 dB SPL (thus, the overall level is approximately 73 dB SPL). The tone spacing corresponded to approximately 1.5 ERBs for moderate sound levels. Given that human cochlear tuning bandwidths are likely narrower than those estimated from simultaneous masking [83], the individual tones would interact only minimally at the auditory periphery. Therefore, their temporal coherence or lack thereof would, at least in part, have to be computed downstream by the central auditory system based on the coherence of neural firing patterns. Each tone was modulated by a random envelope, which was a half-wave rectified and smoothed band-limited (4–24 Hz) noise. The envelope band was chosen so as to overlap with typical speech envelopes. Each stimulus iteration was 4 s long. For the first 1 s, the envelopes of 20 tones were independently realized, and hence temporally incoherent. For the next 1 s, the envelopes of *N* of the tones were the same noise realization, making them maximally coherent, and the remaining 20 − *N* remained uncorrelated. This sequence was repeated a second time, yielding an overall stimulus of 4 s duration. Three different temporal coherence conditions were synthesized with *N =* 6, 12, or 18 tones of the possible 20 being coherent. The particular subset of tones that were coherently modulated and their envelope waveforms were random from trial to trial. Greater values of *N* result in a more salient pop-out of the tone complex subset that is coherent. The trials for the 3 conditions were pseudo-randomly interleaved, with 240 trials presented per condition with an interstimulus interval (ISI) uniformly distributed between 1.2 and 1.3 s. Of the 1.2- to 1.3-s silent periods between stimuli, the last 0.5 s before the onset of a new trial was treated as the baseline period for estimating the noise-covariance matrix for source localization. The MEG recording was performed with participants watching a muted movie without subtitles as the stimuli were presented diotically.

Although our stimulus design was analogous to the stochastic figure–ground (SFG) stimuli used by Teki et al. [32–34], our design deviated from the SFG stimulus in important ways. The SFG stimulus consisted of a cloud of 50-ms-long chords. For each 50-ms window, the set of tone frequencies that formed the chord was either random and uncorrelated with the adjacent chords (when the figure was absent) or was chosen such that a subset of tones followed a coherent time-frequency trajectory (when the figure was present). While this SFG design maintains the overall stimulus amplitude at a roughly constant level, the modulation statistics within some of the frequency bands (i.e., within some tonotopic channels) were allowed to change between the figure-absent and the figure-present portions of the stimulus. This leaves open the possibility that when a figure appears in the stimulus, just the within-tonotopic-channel discontinuities in the modulation statistics can contribute to a neural response. In contrast, the stimuli used in the present study has fixed modulation statistics within each tonotopic channel throughout the length of the stimulus. One consequence of maintaining the within-channel modulation statistics constant and simultaneously increasing the temporal coherence across channels is that the overall amplitude of the stimulus can increase during the coherent portions of the stimulus. However, cochlear processing dictates that the central nervous system does not have access to the overall amplitude and instead is driven by individual tonotopic channels. Accordingly, any neural responses that reflect the overall amplitude would also have to arise from neural computations that use the temporal coherence to combine information across different tonotopic channels. A supplementary behavioral study (S4 Fig) was conducted to validate this reasoning and our modified design.

Audio files of the stimuli are provided in S1–S3 Audios.

### Data analysis and statistical testing

From the left and right auditory cortex ROIs, time courses of evoked responses (i.e., across trial averaged responses) were extracted by simple polarity alignment and averaging. The evoked responses were expected to show an overall onset response at the beginning of the auditory stimulus, and 2 other event-related responses to changes in the temporal coherence that occurred at peristimulus times of *t* = 1 s and *t* = 3 s, marked by white arrows in Fig 1B. The evoked responses for each of the 3 different coherence conditions (*N =* 6, 12, and 18) were obtained by collapsing across the 2 coherence-change events (i.e., by averaging the time course surrounding the event at *t* = 3 s with the time course surrounding the event at *t* = 1 s). The evoked responses derived using this procedure were normalized for each individual by a *z-*scoring procedure relative the 200 ms immediately preceding the coherence-change events. To quantify the M1 component, we average the *z-*scores in the 50- to 150-ms time window. To quantify the M2 component, we average the *z-*scores in the 250- to 450-ms time window.

Because for both the M1 and M2 responses the greatest and most significant main effect of group was for the *N =* 18 coherent tones condition (S2 Fig), and because the M2 component was sometimes difficult to identify in the ASD group, we focused on analyzing the combined M1 + M2 response, computed using the average the *z-*scores in the 50- to 450-ms time window. The combined time window also eliminated the need to be sensitive to variations in individual latencies; while there was no notable group difference in M1 or M2 latencies, undetected latency differences may affect the analysis of individual peaks even in the absence of group differences in latencies.

For the analysis of induced oscillations, spectrograms were estimated from the ROI time courses in each trial and then averaged together. Spectrograms were estimated using the multitaper method for 200-ms-long windows and a time-bandwidth product of 4. This allowed for averaging over estimates from 3 orthogonal maximally concentrated tapers to obtain each time-frequency coefficient [84]. The spectrogram power estimates were then averaged over trials and log transformed. The time axis was then collapsed by averaging, to yield an induced power spectrum in the 5–70 Hz range. Because no systematic differences were seen between left and right hemisphere ROIs in the induced spectra, the responses from the 2 sides were averaged. The induced-oscillation power spectra from each condition and participant in the gamma range (30–70 Hz) were then used for the statistical analysis.

Statistical inference on the MEG ROI data was performed by fitting mixed-effects models to the data and adopting a model comparison approach [85,86]. Two separate sets of models were fit for the evoked-response amplitudes and for the induced spectra. Fixed-effects terms were included for the various experimental factors (i.e., the 3 temporal coherence conditions, the 2 groups, and the group × condition interaction), whereas participant-related effects were treated as random effects. Homoscedasticity of participant-related random effects was not assumed initially, and hence the error terms were allowed to vary and be correlated across the levels of fixed-effects factors. In order to not over-parameterize the random effects, the random terms were pruned by comparing models with and without each term using the Akaike information criterion and log-likelihood ratios [87]. The best-fitting random-effects model turned out to be a single participant-specific random effect that was condition independent (and an overall residual). This random-effect term was used for all subsequent analysis. All model coefficients and covariance parameters were estimated using restricted maximum likelihood as implemented in the lme4 library in R [88]. To make inferences about the experimental fixed effects (i.e., the effect of condition, group, and group × condition interaction), the *F* approximation for the scaled Wald statistic was employed [89]. This approximation is more conservative in estimating type I error rates than the chi-squared approximation of the log-likelihood ratios and has been shown to perform well even with fairly complex covariance structures and small sample sizes [90]. The *p-*values and *F*-statistics based on this approximation are reported.

### Correlations between the neurophysiological measures and behavioral measures

To test whether individual behavioral scores in the ASD group correlated with the neurophysiological measures obtained (evoked responses and induced gamma band activity), we used these neurophysiological measures (from the *N =* 18 condition) as predictors in simple linear regression models, one to predict SRS-SCI score and another to predict SPQ-APS score, for each ASD participant. Thus, the best-fitting linear model was used to obtain the “predicted” behavioral scores exclusively from this combination of neurophysiological measurements. Pearson correlation coefficients between the predicted and the observed behavioral scores are reported to indicate the predictability of behavior from neurophysiology (Fig 3A and 3B).

### Classification by diagnosis

To further assess the relevance of the evoked responses and induced gamma band power to the ASD diagnosis, we used a linear SVM classifier to test whether these neurophysiological metrics allow for blind classification of individuals into their corresponding groups. Specifically, the individual *z-*scores for gamma band power and normalized evoked responses (both for the *N =* 18 coherent tones condition) were used as the predictive features. The full dataset of *N =* 47 participants was randomly partitioned into a training sample containing 90% of the participants and a test sample containing the remaining 10%. The accuracy of the optimal linear SVM classifier obtained from the training sample was then assessed by comparing the predicted class labels to the actual class labels in the test sample. To obtain the mean and standard error of the classification accuracy, this 90%–10% train–test split was repeated 50 times with replacement. The classifier had an accuracy of 83% ± 3% for group classification (Fig 3A) across the 50 train (90%)–test (10%) splits.

### Ethics statement

The study was conducted in accordance with the principles expressed in the Declaration of Helsinki. Parents provided written informed consent according to protocols approved by the Massachusetts General Hospital Institutional Review Board (IRB protocol #2005P001768). Participants provided assent in addition to parent consent.

## Supporting information

S1 Audio Auditory file of stimulus example for the *N =* 6 coherent tones condition. The attached auditory file is an example stimulus heard by the participants.
(WAV)

S2 Audio Auditory file of stimulus example for the *N =* 12 coherent tones condition. The attached auditory file is an example stimulus heard by the participants.
(WAV)

S3 Audio Auditory file of stimulus example for the *N =* 18 coherent tones condition. The attached auditory file is an example stimulus heard by the participants.
(WAV)

S1 Fig Evoked responses by hemisphere. (A) The averaged amplitude of the evoked responses for each condition (*x*-axis), over the 50-ms to 450-ms time window, for each hemisphere (left = solid line, right = dashed line), for the TD group. (B) Same as (A), for the ASD group. There were no hemispheric differences in either group (*p* = 0.21). Underlying data can be found on Zenodo (doi: 10.5281/zenodo.5823656).
(EPS)

S2 Fig M1 and M2 components of the evoked responses. (A) The M1 component (50–150 ms) of the evoked responses from Fig3A–3C, by condition, for both groups. The group difference was only significant for the *N =* 18 coherence tones condition. (B) Same as (A), for the M2 component of the response (250–450 ms). The group difference was significant for both the *N =* 12 and *N =* 18 coherent tones conditions, with differences getting larger with coherence. For main effect of group: *F*(1,45) = 9.65, *p* = 0.003; for group × condition interaction: *F*(2,90) = 3.4, *p* = 0.03. Underlying data can be found on Zenodo (doi: 10.5281/zenodo.5823656).
(EPS)

S3 Fig SPQ-APS and age. (A) SPQ-APS plotted versus age, for the ASD group. (B) Same as Fig 4C, just without the age correction: The behaviorally assessed auditory processing subscore, SPQ-APS, uncorrected for age, plotted against the same score predicted using the individual evoked responses and induced gamma band activity. Underlying data can be found on Zenodo (doi: 10.5281/zenodo.5823656).
(EPS)

S4 Fig Data from a behavioral experiment testing the contribution of across-channel relationships versus the contribution of overall stimulus amplitude, for the detection of the temporally coherent figure. Figure detection data were collected from *N =* 6 adult participants with 2 coherence levels (4 coherent tones or 6 coherent tones) that were near the detection threshold. (A) The acoustic time course of an example stimulus where the coherently modulated tones were closer in frequency spacing. (B) The acoustic time course of an example stimulus where the coherently modulated tones were wider in spacing, and interspersed with incoherently modulated tones. Because the number of coherently modulated tones is the same in (A) and (B), the amplitude increase is the same for both categories of stimuli. (C and D) The spectrograms of the stimuli in (A) and (B), respectively. (E) Detection accuracy dropped significantly when the frequency separation was high (6.3 ERBs or 5.7 ERBs—[B and D]), compared to when the frequency separation was smaller (1.5 ERBs—[A and C]), despite both stimuli producing the same increase in overall stimulus amplitude during the coherent portions. This demonstrates that it is the coherence relationships across different tonotopic channels, and not the overall amplitude, that is most likely to be the primary driver of figure detection, as expected. Underlying data can be found on Zenodo (doi: 10.5281/zenodo.5823656).
(EPS)

S5 Fig ICSS-I scores and MEG gamma band activity. As with the evoked responses, no significant correlations were observed between ICSS-I score, which measures overall ability to inhibit attention to unwanted stimuli, and induced gamma band activity. Underlying data can be found on Zenodo (doi: 10.5281/zenodo.5823656).
(EPS)

## Abbreviations

ASD: autism spectrum disorder
ICSS-I: NEPSY-II–Inhibition Inhibition Contrast Scaled Score–Inhibition
MEG: magnetoencephalography
MRI: magnetic resonance imaging
ROI: region of interest
SPQ-APS: Sensory Profile Questionnaire–Auditory Profile Subscale
SRS-SCI: Social Responsiveness Scale–Social Communication and Interaction
SVM: support vector machine
TD: typically developing
